# Best practices for implementing ChatGPT, large language models, and artificial intelligence in qualitative and survey-based research

**DOI:** 10.1016/j.jdin.2023.10.001

**Published:** 2023-10-08

**Authors:** Jonathan Kantor

**Affiliations:** Department of Dermatology, Center for Global Health, and Center for Clinical Epidemiology and Biostatistics, Perelman School of Medicine at the University of Pennsylvania, Philadelphia, Pennsylvania; Florida Center for Dermatology, St Augustine, Florida

**Keywords:** AI, annotation, artificial intelligence, ChatGPT, factor analysis, focus group, Google Bard, GPT-4, large language models, LLM, medical education, Microsoft Bing, natural language processing, NLP, practice management, prompt engineering, QDA, qualitative research, qualitative data analysis software

Large language models (LLMs), propelled by the popularity of OpenAI’s ChatGPT, are being increasingly investigated for research and clinical applications in science and health care.^1^, ^2^, ^3^ Yet in many ways, the nature of generative artificial intelligence models may also be well suited for qualitative researchers—those investigating the complexity of human behavior, choices, attitudes, and preferences. Given the models’ ability to immediately synthesize vast quantities of text coupled with their potential to winnow a range of disparate inputs into cohesive themes, they may present significant opportunities for qualitative researchers eager to diversify and deepen their analyses.

Qualitative researchers may use LLMs in several ways: hypothesis generation, data analysis, coding and thematic analysis, natural language processing, mixed methods research, data triangulation, literature review and synthesis, and writing. In hypothesis generation, LLMs can explore data for associations and correlations, increasing the speed with which researchers can assess data while also presenting novel themes that may not be obvious to human researchers. For data analysis, LLMs can be used to extract insights, trends, and patterns from data and help better define research variables, thematic constructs, and theoretical frameworks. Coding is a notoriously laborious process; while some qualitative data analysis software has included autocoding in the past, LLMs far surpass these native abilities, making such existing features essentially outdated. Still, given that the coding process is itself a way for the researcher to engage with the data and refine hypotheses, it is critical to avoid overautomating this approach in the name of efficiency thereby undermining the researcher’s ability to engage with their data adequately and deeply. Natural language processing can be useful for sentiment analysis and as an adjunct to the researcher’s existing sentiment analysis approaches, and it may be interesting to use the LLM as an additional set of virtual eyes to gain additional insights beyond those developed by the researcher.

In mixed methods research, LLMs could help facilitate the integration of qualitative and quantitative data, highlighting opportunities for complex multilayered analyses, and ultimately helping researchers move beyond the how and why. For literature reviews, LLMs can help summarize the existing literature and may expedite the development of summary measures, particularly for the text-rich manuscripts common in qualitative research. Finally, the process of writing is central to qualitative research, and although LLMs certainly cannot compete with experienced researchers in the construction of effective prose, they can be used to augment the drafting process, offering a springboard for content generation.

There are also some potential risks of broad LLM adoption: hallucination, errors and inaccuracies, bias amplification, and of course the dreaded atrophy of fundamental skills.^4^^,^^5^ Further, given that the goal of qualitative research is often explanatory or exploratory, the black box nature of LLMs may be a significant liability. Most importantly, LLMs should always be used as an adjunct to, rather than a replacement for, a researcher’s overall skillset and intuition. That said, LLMs show significant promise for qualitative researchers, and thoughtful engagement, rather than strict containment, maybe the wisest path forward.

## Conflicts of interest

None disclosed.
